# Psychosocial recovery after serious injury

**DOI:** 10.3402/ejpt.v5.26516

**Published:** 2014-12-09

**Authors:** Meaghan O'Donnell

**Affiliations:** Department of Psychiatry, University of Melbourne, Melbourne, Australia

**Keywords:** injury, psychosocial recovery, transdiagnostic, early intervention, anxiety, depression, PTSD, agoraphobia, substance use

## Abstract

**Background:**

The 2010 iteration of the Global Burden of Disease statistics (Murray et al., 2012) points to the growing impact of injury and highlights the mounting burden of psychiatric disorder. It is essential to examine the intersection between these two contributors to disease burden.

**Methods:**

The Australian Injury Vulnerability Study collected data of over 1,000 injury patients from their initial hospitalization to 6 years post-injury. Structured clinical interviews were used to diagnose psychiatric disorder and self-report measures for disability and symptom severity.

**Results:**

A wide range of psychiatric disorders developed following injury, which included posttraumatic stress disorder, agoraphobia, depression, and substance use disorders (Bryant, O'Donnell, Creamer, Silove, & McFarlane, 2010). Although prevalence rates for these disorders were generally consistent over time, examination of trajectory data showed that different people had the disorders at different times. Importantly, the data showed that early anxiety, depression, and PTSD symptoms played a significant role in the development of long term disability after injury (Carty, O'Donnell, Evans, Kazantzis, & Creamer, 2011; O'Donnell et al., 2013).

**Conclusions:**

These data support the view that transdiagnostic models for early intervention may be required to address the complex psychiatric disorder trajectories that develop after injury.
